# Supplementary material for: Chronologically distributed transfection improves AAV2 and AAV2/8 capsid filling and reveals assembly schedule divergence
Source: Mol Ther Methods Clin Dev. 2025 Oct 4;33(4):101610. doi: 10.1016/j.omtm.2025.101610 (PMC12554105; doi:10.1016/j.omtm.2025.101610)
Supplement: Document S1. Figures S1 and S2 [file mmc1.pdf]

OMTM, Volume *33*

Supplemental information

Chronologically distributed transfection improves AAV2 and AAV2/8 capsid filling and reveals assembly schedule divergence

Qiantong Chen, Chae Hyon Lee, Robert Whitfield, and Darren N. Nesbeth

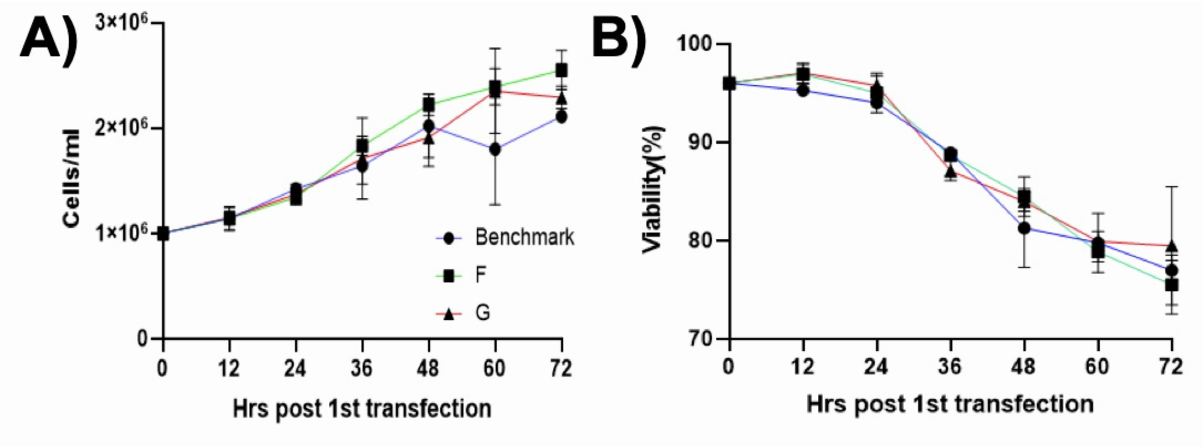

Figure S1. Growth and viability of HEK293F cells after T1 transfection for chronologies Bmk, F and G. From the T1 transfection (Figure 1.3) of chronologies Bmk, F and G, HEK293F cells were analysed with respect to A) Viable cell density (Cells/mL) and B) Viability (%). Error bars represent standard deviation from two biological repeats. Symbol key within plot A also applies to plot B. For transfections chronologies F and G, a second transfection, T2, was performed 20 hours post T1.

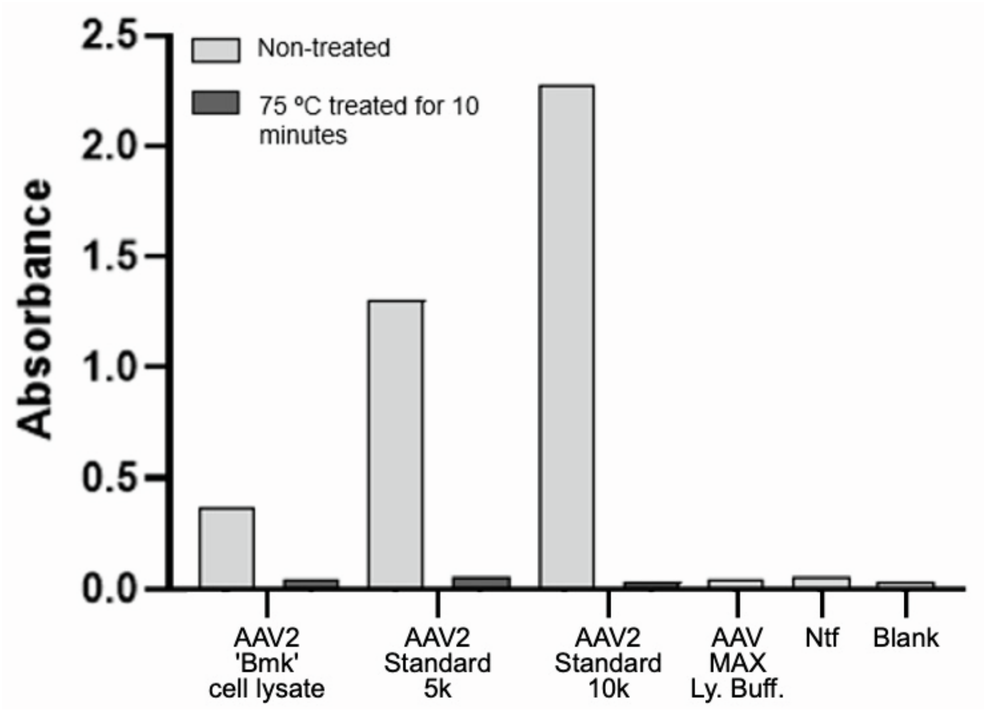

Figure S2. Temperature sensitivity of ELISA signal arising from samples containing AAV2 capids. Samples from benchmark ('Bmk') transfection of HEK293 cells and of standard AAV2 material (Progen, 66V021) were analysed using a Genscript 'AAV2 Titer Capsid ELISA Kit'. Using Sample dilution buffer (Genscript, G1-60) from the ELISA kit as diluent, the cell lysate sample was diluted 1:625 and the AAV standard sample diluted 1:5,000 ('5k') or 1:10,000 ('10k'). Gibco™ AAV-MAX Lysis Buffer (Fisher Scientific, 17331899), lysate from untransfected cells ('Ntf'), and the Sample dilution buffer ('Blank') (Genscript, G1-60), were analysed as controls. All samples were either non-treated (light gray) or placed in a hot block (Bio-Rad, model T100) set at 75°C for 10 minutes (dark gray). Procedures performed in singlet.
