# Supplementary material for: Chronologically distributed transfection improves AAV2 and AAV2/8 capsid filling and reveals assembly schedule divergence
Source: Mol Ther Methods Clin Dev. 2025 Oct 4;33(4):101610. doi: 10.1016/j.omtm.2025.101610 (PMC12554105; doi:10.1016/j.omtm.2025.101610)
Supplement: Document S2. Article plus supplemental information [file mmc2.pdf]

Molecular Therapy
Methods & Clinical Development
Original Article

Chronologically distributed transfection improves AAV2 and AAV2/8 capsid filling and reveals assembly schedule divergence

Qiantong Chen,[1,2] Chae Hyon Lee,[1] Robert Whitfield,[2] and Darren N. Nesbeth[1]

[1]Department of Biochemical Engineering, University College London, Bernard Katz Building, London WC1E 7JE, UK; [2]Cytiva Life Sciences, Amersham Place, Little Chalfont, Amersham HP7 9NA, UK

Adeno-associated virus (AAV) gene therapy vectors often suffer from low capsid filling, resulting in high proportions of empty capsids that reduce efficacy and complicate manufacturing processes. This study investigates whether chronologically distributed transfection could improve capsid filling for AAV2 and AAV2/8 serotypes. We used an empirical approach to test different transfection chronologies by varying the timing of Helper, RepCap, and Payload plasmid delivery across two time points, T1 and T2 (24 and 44 h post-seeding, respectively). Our results revealed distinct serotype-specific responses to altered transfection chronologies, with AAV2/8 production being robust to a broader range of chronologies than AAV2. All non-standard chronologies reduced physical and biological titers. Notably, T1 transfection with Helper and Payload plasmids, followed by RepCap plasmid at T2, increased capsid filling efficiency by approximately 7.5-fold for both AAV2 and AAV2/8. This finding provides empirical support for a temporal misalignment hypothesis, whereby suboptimal AAV capsid filling results from capsid assembly occurring before peak genome replication. Our study demonstrates a re-scheduled transfection procedure that can enhance AAV production outcomes and reveals fundamental differences in assembly dynamics between serotypes. These insights contribute to understanding AAV assembly mechanisms and offer a novel method for process development in gene therapy manufacturing.

INTRODUCTION

Recombinant adeno-associated virus (AAV) is a leading gene therapy tool, affecting long-term transgene expression in both dividing and non-dividing therapeutic target cells.[1] AAV serotype 2 (AAV2) has strong tropism for neurons and retinal cells, making it particularly suitable for central nervous system and ocular gene therapies.[2] AAV2/8 exhibits robust liver tropism and ability to cross the blood-vessel barrier, enabling efficient systemic delivery for liver-directed gene therapies.[3]

AAV production methods typically yield a high proportion of empty capsids lacking the genome encoding the therapeutic transgene, sometimes exceeding 90% of total viral particles.[4] Empty capsids can burden manufacturing capacity, complicate downstream purification, and reduce efficacy by triggering immune responses and competing with full capsids for cellular receptors.[5]

Typical AAV production is via transient, three-plasmid (Figure 1.1) co-transfection of HEK293-based cells.[6] The "Payload" plasmid encodes the therapeutic transgene flanked by inverted terminal repeats (ITRs). The "RepCap" plasmid encodes AAV Rep and Cap genes, which direct production of four and six proteins, respectively.[7] ITRs are omitted from the RepCap plasmid to prevent formation of replication-competent AAV virus.[8] The "Helper" plasmid encodes a subset of adenoviral genes that support AAV production but are insufficient for replication-competent adenovirus formation, to ensure safety.[6,9] After three-plasmid co-transfection (Figure 1.2), proteins encoded by the Helper and RepCap plasmids bring about targeted replication of the "payload genome" segment of the Payload plasmid, spanning the two ITRs and the region they flank. These proteins also bring about formation of viral capsid particles and insertion of the payload genome into those particles.[10,11]

The precise nature and schedule of the intracellular molecular events that define AAV capsid assembly and genomic filling remains incompletely understood. The potential gene therapy benefits of improving capsid filling with intended genomic payload (the "empty:full" ratio) motivate ongoing investigation into the events that define AAV particle synthesis and assembly within transiently transfected host cells.

The temporal misalignment hypothesis

Separation of RepCap genes onto the RepCap plasmid and ITRs onto the Payload may contribute to the lower packaging efficiency observed with recombinant AAV compared to the wild type.[12–14] Spatial dispersion of RepCap and Payload plasmids within transfected host cells may disrupt the regulatory dynamics of AAV gene

Received 17 July 2025; accepted 1 October 2025;
https://doi.org/10.1016/j.omtm.2025.101610.

Correspondence: Darren N. Nesbeth, Department of Biochemical Engineering, University College London, Bernard Katz Building, London WC1E 6BT, UK.
E-mail: d.nesbeth@ucl.ac.uk

© 2025 The Author(s). Published by Elsevier Inc. on behalf of The American Society of Gene and Cell Therapy.
This is an open access article under the CC BY license (http://creativecommons.org/licenses/by/4.0/).

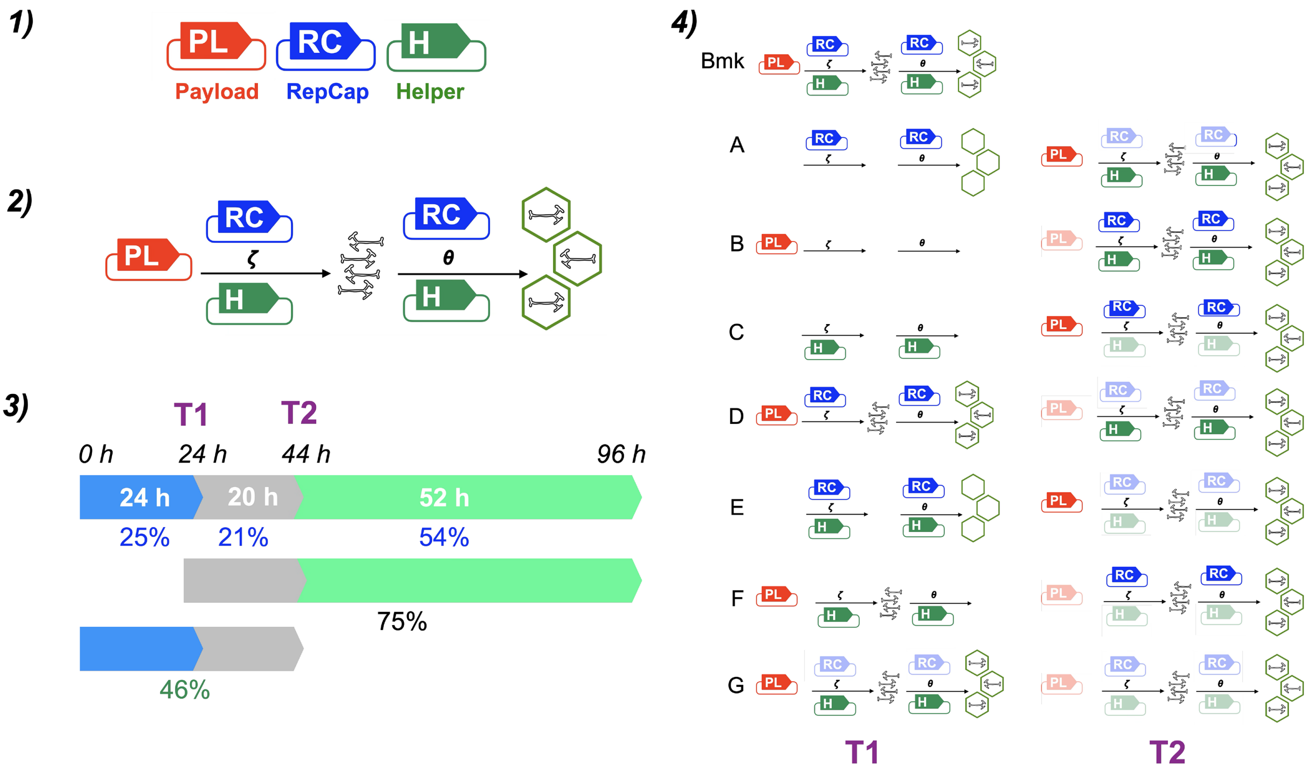

Figure 1. Illustrative diagrams of AAV genome and capsid production and assembly plasmids and transfection timelines

(1) Simplified diagrams of Payload (red), RepCap (blue), and Helper (green) plasmids. (2) Occam schema of zeta (ζ) procedures, leading to correct payload genome production, and theta (θ) procedures, which require at least one payload genome in order to commence, for production of capsids correctly loaded with payload genomes. (3) Diagram of time points for the transfection chronologies used in this study. At 0 h, host cells are seeded, with transfection 1 (T1) 24 h post-seeding, T2 44 h post-seeding, and harvest 96 h post-seeding. The duration of time periods between those four events are also depicted in hours and as a percentage of the 96 h total. (4) Graphical depictions of the eight transfection chronologies used in this study and their predicted consequences. For chronologies A–F the following applies: plasmids are depicted as being partially opaque at T2 if they were present at T1. Payload genomes or correctly packaged capsids are absent if their formation is not conventionally understood to be likely or possible. A prediction of empty capsid formation is depicted for the T1 θ procedures whenever payload genome production is predicted to be impossible for the T1 ζ procedures. For chronology G, the same rules apply except for the RC plasmid, which is depicted as partially opaque at both T1 and T2, as half the RC plasmid mass of the Bmk transfection was used in both transfections. For Bmk there is only a single transfection, T1, and no predictions are made for the abundance of any elements at T2, hence no diagrams.

expression, impacting the sequence and duration of events that occur in wild-type AAV[15] and triple transfection.[16] Nguyen et al. developed a general mechanistic model of recombinant AAV assembly that predicted an early peak of capsid synthesis followed by a later peak of payload replication.[17] They interpreted this as a temporal misalignment between capsid assembly and DNA replication, which may contribute to the high "empty:full" ratio.

This hypothesis was supported by observations reported by Lee et al.[18] and Lu et al.,[19,20] who engineered separate inducible control into a selection of Helper and RepCap plasmid genes and then stably integrated them into HEK293 cells for AAV2 production. Ohba et al.[21] engineered inducible control into a version of the Cap gene removed from the context of the RepCap plasmid and achieved maximal improvement in AAV2 capsid filling by delaying Cap induction until 12 h after standard three-plasmid transient transfection. Srinivasan et al.[22] distributed the standard three-plasmid transient transfection over two or three separate transfections, with each individual transfection having one-half or one-third the usual plasmid mass, respectively. Their experimental observations were used to refine a model that again supported a temporal misalignment hypothesis, in which AAV5 capsids formed at an early stage achieve maximal filling only when accompanied by an optimal expression timing and abundance of proteins encoded by the Rep gene.

Transfection chronologies to test the TMA hypothesis and improve AAV production

The consistent set of results and simulations from the above reports strongly support temporal misalignment (TMA) as a hypothesis for packaging inefficiency in AAV production from the standard triple-plasmid transient transfection. Specifically, TMA predicts an early peak of capsid assembly and a later peak of payload replication as a cause of low capsid filling efficiency. Therefore, we sought to apply a new test of the TMA hypothesis by performing a series of novel, chronologically distributed single-, double-, and three-plasmid HEK293F cell transfections. We anticipated that empirical screening

Table 1. Transfection chronologies used in this study

Reaction code	Transfection 1 (T1) plasmids(s)			Transfection 2 (T2) plasmid(s)		
Benchmark (Bmk)	pRepCap	pHelper	pPayload	–	–	–
A	pRepCap	–	–	–	pHelper	pPayload
B	–	–	pPayload	pRepCap	pHelper	–
C	–	pHelper	–	pRepCap	–	pPayload
D	pRepCap	–	pPayload	–	pHelper	–
E	pRepCap	pHelper	–	–	–	pPayload
F	–	pHelper	pPayload	pRepCap	–	–
G	(pRepCap)/2	pHelper	pPayload	(pRepCap)/2	–	–

Overview of transfection chronologies investigated in this study. The benchmark (BmK) procedure introduces all three plasmids (pRepCap, pHelper, and pPayload) in a single transfection at time point T1 (Figure 1.3). Chronologies A–F explore various combinations of plasmid delivery timing and grouping across two transfections (T1 and T2). Chronology G is the same as Bmk, except that half the RepCap plasmid mass is used in the T1 transfection and the remaining half used in a T2 transfection.

of seven different transfection chronologies (Table 1) would reveal a pattern of viral filling activities that either aligns with or conflicts with TMA, thereby providing valuable insight into the role of the timing of RepCap, Payload, and Helper-encoded gene functions in AAV yield and filling performance. Furthermore, by using only standard plasmids and reagents, we considered that any transfection chronologies that might improve upon the performance of standard methods would be readily implementable by the wider gene therapy bioprocess community, with no requirement for plasmid re-design.

We performed chronologically distributed, three-plasmid transfection reactions, with transfection being performed 24 ("T1") and 44 h ("T2") after seeding of host cells and with viral harvest 96 h after seeding (Figure 1.3). The chronologies we tested included all possible combinations and sequences of 2-plasmid and 1-plasmid transfections. We combined this empirical design with a rational schema, described below, of how the combined actions of the three plasmids lead to AAV particle formation (Figure 1.4) plus three reasonable assumptions regarding AAV production by transient transfection.

Our first assumption was that, over time, intracellular plasmid concentration and plasmid gene expression reduce from a post-transfection maximum. Such decreasing plasmid abundance has been observed to result from plasmid degradation[23] and plasmid dilution by cell division.[24] Decreasing expression of plasmid-encoded genes, after a post-transfection maximum, has been reported in the context of mammalian cell transient transfection for production of AAV,[25] lentivirus,[26] virus-like particles,[27] and recombinant protein.[28]

Our second assumption was that Payload plasmids cannot be packaged *in totum* into AAV capsids. Payload plasmids are covalently closed circular double-stranded DNA (dsDNA) molecules, thus with no exposed 5′ phosphate or 3′ hydroxyl groups. A typical payload plasmid, such as the pAAV-GFP plasmid used in this study, is 5.7 kilo base pairs in size, totaling approximately 11,400 nucleotides. This far exceeds the typical AAV packaging capacity of 4.7 kilo bases of single-stranded,[29] linear DNA (approximately 4,700 nucleotides). Furthermore, when others have surveyed the range of DNA molecules packaged into a population of AAV virus particles,[30–33] no *in totum* Payload plasmids were detected.

Our third assumption was that, in the context of multiple (2–3) transfections being performed on the same group of HEK293 cells, prior transfection procedures do not significantly compromise the performance of subsequent transfections in terms of functional transgene expression. This assumption is supported by reports by Cervera et al.[34] and Riedl et al.[35] when using multiple HEK293 transfections to increase the yield of plasmid-encoded recombinant protein.

RESULTS

"Occam schema" as an abductive reasoning aid to predict transfection performance

In recombinant AAV production, the Payload plasmid is the essential initial template substrate for nicking and DNA replication to produce multiple copies of the linear, ITR-flanked, recombinant payload genome (referred to henceforth as "payload genome(s)" for brevity). It is understood that a combination of host cell functions, and RepCap and Helper plasmid gene functions, all contribute to this process.[7,30,36]

In Figure 1.2, we propose an "Occam schema" of this plasmid-based AAV production, as an abductive reasoning aid to guide our predictions regarding the likely impacts of the seven different transfection chronologies we have trialed. In this schema, we refer collectively to all the combined functions that lead to payload genome production as "zeta procedures," with the Greek zeta symbol, ζ, as a crude graphical representation of a single-stranded, linear DNA payload genome. Once at least one payload genome has been produced, RepCap and Helper plasmid gene function can then support production and correct filling of capsids, without the absolute requirement for the presence of the Payload plasmid. In Figure 1.2, we refer to these functions that lead to production of correctly packaged capsids as "theta procedures," with the Greek theta symbol, θ, crudely representing a correctly filled capsid. Our proposed schema makes no prediction of correctly packaged AAV production dynamics and is

not intended as a mechanistic model. However, we use it here as an abductive reasoning aid to guide our predictions regarding the likely impacts of the seven different transfection chronologies we have trialed.

Before performing the standard "Benchmark" (Bmk) transfection and seven alternative transfection chronologies, we used the Occam schema of conventional AAV production (Figure 1.2) as a framework for our predictions of the likely impacts of the seven chronologies. We set out these predictions below and provide graphical summaries in Figure 1.4. The uppermost row of Figure 1.4 shows the Bmk transfection chronology, with all three plasmids present in a single transfection performed 24 h after cell seeding (T1). As all three plasmids are present, ζ- and θ-procedures are both theoretically supported throughout the transfection period. With respect to the TMA hypothesis, we can interpret the Bmk transfection yield performance, in terms of payload genomes, capsids, and correctly filled capsids, as being characteristic of the proposed misalignment. Changes to performance against any of these metrics can be interpreted as improvement or exacerbation of the misalignment of assembly events.

Transfection chronologies A, B, and C

In Figure 1.4, the row labeled "A" (Figure 1.4A) depicts the likely impacts of transfection chronology A (Table 1). Only the RepCap plasmid is present in a first transfection performed 24 h after cell seeding (T1). Helper and Payload plasmids are only provided in a second transfection performed 44 h after cell seeding (T2). In the context of the Occam schema, no ζ- or θ-procedures are possible for the 44 h between seeding and T2. During this period, a level of capsid particles may be assembled, albeit at a very low level due to the absence of Helper, but correct capsid filling will be impossible due to the absence of Payload plasmid. For the T2 graphic in Figure 1.4A, we have faded the RepCap plasmid, as we predict that a decrease in RepCap plasmid gene expression during the 72-h period after T1 is likely to precede the decrease in Payload and Helper plasmid gene expression during the 52-h period after T2 (see Figure 1.3 for timeline). For chronologies B–F, the graphics for plasmids used in T2 transfections are faded for the same rationale.

Given that ζ and θ procedures are impossible for 46% of the post-seeding period for chronology A, as opposed to 25% for the Bmk chronology, we predict an overall reduction in volumetric genome yield performance for transfection A compared to Bmk. Such a result would also be in line with TMA, as, in effect, transfection A exacerbates the misalignment between early capsid production peak and a later payload genome production peak.

For transfection chronology B (Table 1; Figure 1.4B), only Payload plasmid is present for T1 and only Helper and RepCap plasmids for T2. ζ- and θ-procedures are not possible for the 44 h between seeding and T2, and as a result, we predict no payload genome or capsid production during that period. We would expect that, for a fixed amount of RepCap and Helper plasmid, the total amount of Payload plasmid template present would delimit the final yield of payload genomes. As such, compared to the Bmk chronology, for chronology B RepCap and Helper plasmid gene, functions are likely to have a lesser amount of Payload plasmid to use as template for ζ-procedures. Overall, we would again predict a reduced genome yield for chronology B and that this would be consistent with TMA.

Transfection chronology C (Table 1; Figure 1.4C) has only Helper plasmid for T1, and as such, we suggest the same yield predictions for chronologies A and B would apply, as this plasmid alone is insufficient for payload genome or capsid production. Overall, for transfection chronologies A–C, the T1 transfection is a single plasmid and therefore insufficient to support ζ- or θ-procedures.

Transfection chronology D

For transfection chronology D (Figure 1.4D), Payload and RepCap plasmids are present for T1, and Helper plasmid is only provided at T2. We would predict that ζ- and θ-procedures can take place within the 20-h T1-T2 intervening period, but to a reduced level of production compared to Bmk, due to the absence of Helper plasmid for this period. Any rescue of production from T2 forward, with the addition of Helper plasmid, would be balanced against the relatively decreasing contribution of Payload and RepCap plasmid gene function over time. In the context of the TMA hypothesis, chronology D could be predicted to either (1) leave net yields unchanged or (2) further exacerbate misalignment of capsid and payload genome production, depending on which of these processes is more sensitive to the presence of Helper plasmid gene function.

Transfection chronology E

In transfection chronology E (Figure 1.4E), Helper and RepCap plasmids are present for T1, and Payload plasmid is provided at T2. We would predict that neither ζ- nor θ-procedures can take place within the T1-T2 intervening period, with only empty capsid production possible. From T2 forward, the addition of Payload plasmid would enable ζ- and θ-procedures to commence, against a background of decreasing relative abundance of Helper and RepCap plasmid gene function over the 52-h, post-T2 period. Chronology E could be firmly predicted to exacerbate the misalignment of capsid and payload genome production as part of the TMA hypothesis.

Transfection chronology F

For transfection chronology F (Figure 1.4F), Helper and Payload plasmids are present for T1, and RepCap plasmid is provided at T2. Nash et al.[37] and van Lieshout et al.[38] report that Helper plasmid and the intracellular environment are sufficient to support a level of payload genome replication from Payload plasmid template. As such, we would predict that ζ-procedures can take place within the 20-h T1-T2 intervening period. From T2, the addition of RepCap plasmid enables both ζ- and θ-procedures to commence. Chronology F may remedy the misalignment of capsid and payload genome production of the TMA hypothesis and mimic the wild-type AAV

Table 2. Component volumes used in transfections in this study

		T1				T2			
		DNA mixture		PEI mixture		DNA mixture		PEI mixture	
	Transfection chronology	µL plasmid	µL Opti-MEM	µL PEI	µL Opti-MEM	µL plasmid	µL Opti-MEM	µL PEI	µL Opti-MEM
AAV2 (10 mL)									
1	Bmk	54.8	445.2	15	485	0	0	0	0
2	A	4.3	34.6	4.1	131.2	50.5	410.6	10.9	353.8
3	B	4.4	35.4	2.4	77.8	50.4	409.8	12.6	407.2
4	C	46.2	375.2	8.5	276	8.6	70	6.5	209
5	D	8.6	70	6.5	209	46.2	375.2	8.5	276
6	E	50.4	409.8	12.6	407.2	4.4	35.4	2.4	77.8
7	F	50.5	410.6	10.9	353.8	4.3	34.6	4.1	131.2
8	G	52.7	427.9	13	419.4	2.1	17.3	2	65.6
AAV2/8 (1 mL)									
9	Bmk	5.1	44.9	1.5	48.5	0	0	0	0
10	A	0.4	3.3	0.4	12.6	4.7	41.6	1.1	35.9
11	B	0.6	5.4	0.3	9.3	4.5	39.5	1.2	39.2
12	C	4.1	36.2	0.8	26.6	1	8.7	0.7	21.9
13	D	1	8.7	0.7	21.9	4.1	36.2	0.8	26.6
14	E	4.5	39.5	1.2	39.2	0.6	5.4	0.3	9.3
15	F	4.7	41.6	1.1	35.9	0.4	3.3	0.4	12.6
16	G	4.9	43.2	1.4	42.2	0.2	1.7	0.2	6.3

Component volumes used in DNA transfection reactions across one (T1) or two time points (T1 and T2) for serotypes and production volumes indicated. Each reaction condition (Bmk through G) consists of a DNA mixture, containing plasmid DNA diluted in Opti-MEM medium, and a polyethylenimine (PEI) mixture, containing PEI diluted in Opti-MEM medium. All volumes are recorded in microliters (µL). Volumes indicated are per individual transfection. For all transfections, a "Master Mix" was made with a sufficient volume for a given number of repeats plus one more. As such, the smallest volume actually pipetted was 0.66 µL using a Gilson P2 pipette.

dynamics,[17] by preventing any capsid assembly and Rep synthesis during the first 44 h of cultivation post-seeding. An increase in an AAV production metric resulting from chronology F may therefore further support the TMA hypothesis, as well as represent a potential route to improving AAV upstream bioprocessing.

Transfection chronology G

In transfection chronology G (Figure 1.4G), Helper and Payload plasmids are present for T1, and one-half the mass of RepCap plasmid used in the Bmk chronology is provided at T1 and T2. In this case, we have depicted the RepCap plasmid graphic as being faded at both these time points to depict this use of half-Bmk mass. We predict that both ζ- and θ-procedures can take place from T1 onward, with the relative abundance of Helper and Payload plasmid gene function declining at a point after T1, while RepCap plasmid abundance has two peaks within the 72-h period post-T1. We again anticipate that this chronology may remedy capsid and payload genome production misalignment, by distributing the abundance of RepCap over time compared to Bmk chronology (Figure 1.4, uppermost row). As with chronology F, if chronology G increases performance against an AAV production metric, this would be consistent with the TMA hypothesis and may be a promising method for AAV production.

Influence of transfection chronology on production of nuclease-protected viral genomes

To lend this study maximum relevance for the gene therapy bioprocess community, we performed all yield-based experimentation on AAV2 and AAV2/8 serotypes at the lowest scale compatible with statistically rigorous observations. For AAV2, this minimal production scale was 10 mL and for AAV2/8 it was 1 mL. We anticipated that using these scales would lower the barriers to other groups, wishing to expand or replicate the results we report. Process development benefits significantly from operating at the smallest practical scale, primarily due to resource efficiency and the ability to explore a broader experimental space. By minimizing the scale, the consumption of costly raw materials such as plasmid DNA, transfection reagents, and cell culture media is substantially reduced, enabling more experimental conditions to be tested within the same budget. This increased throughput is particularly valuable during the initial phases of process development where multiple parameters need to be screened and optimized.

We used standard bench-scale procedures for AAV2 and AAV2/8 production by transient transfection and analysis of crude lysate using quantitative PCR (qPCR) (see materials and methods; and Tables 1 and 2). This standard qPCR procedure included an

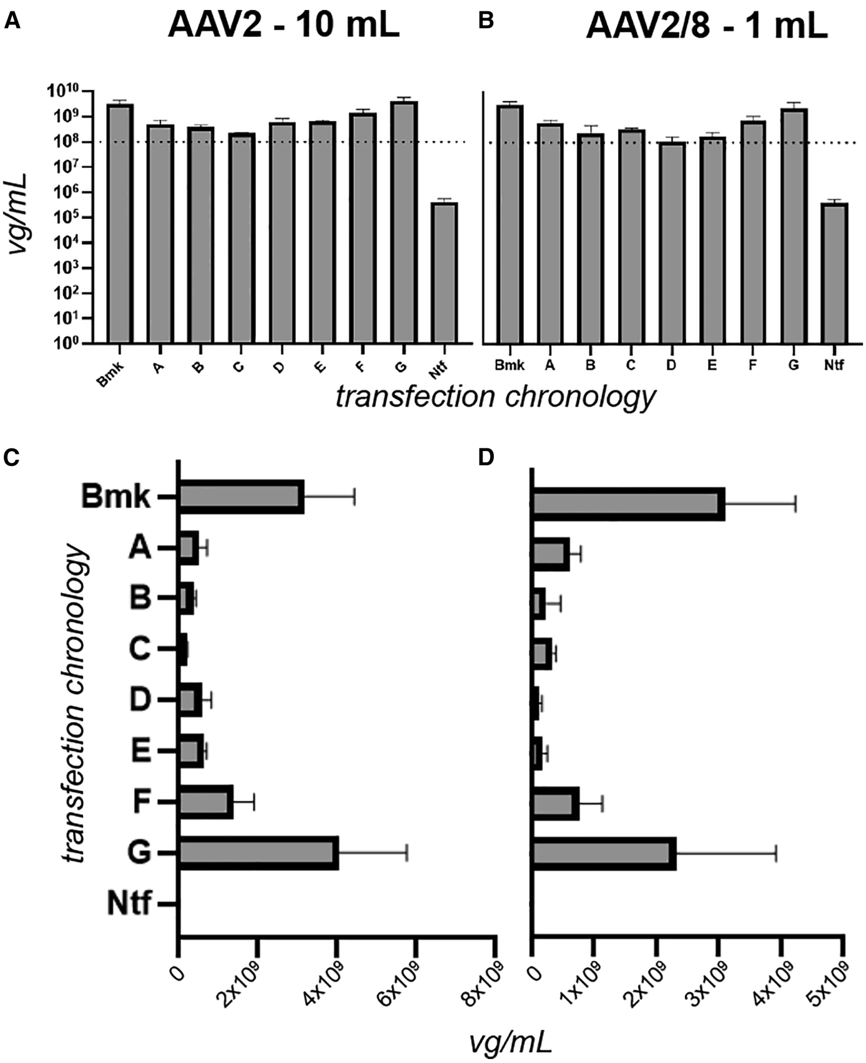

Figure 2. AAV2 and AAV2/8 viral genome yield performance as a function of transfection chronologically

Viral genome titers were determined by qPCR for AAV2 and AAV2/8 crude lysates arising from the transfection chronologies illustrated in Figure 1.4 by letter code, at 10 mL and 1 mL scales, respectively. Plots (A) and (C) show AAV2 results on log and linear scales respectively, while plots (B) and (D) show AAV2/8 results on log and linear scales, respectively. Crude lysate from non-transfected cells (Ntf) were used as qPCR template and interpreted as the limit of quantitation (LOQ) for the assay. The dotted line highlights the lowest yield achieved by any transfection chronology. Error bars represent standard deviation of three biological replicates.

incubation with nuclease in order to degrade all DNA present in soluble crude cell lysate material, including unpackaged payload genomes.[39,40] We therefore reasoned that all genomes quantified are likely to have been protected by association with capsids capable of sequestering them from nuclease-based degradation.

All transfection chronologies achieved or exceeded a minimum level of 10^8 vg/mL (Figures 2A and 2B). These values were three orders of magnitude above the 10^5 vg/mL limit of quantitation (LOQ) for the assay. For both serotypes, chronologies A–E represented a lower plateau of vector genome (vg) yield performance, compared to F and G (Figures 2C and 2D). This is particularly notable when comparing chronologies D and F, where the T1 presence of Helper plasmid (chronology F) is associated with greater vg yield than the T1 presence of RepCap plasmid (chronology D). This is counter to expectations of a greater role for RepCap in payload genome replication and also supports the concept of plasmid presence and cellular protein functions having the ability to "prime" cells for subsequent virus production.[41,42]

Payload genome production is possible for 54% of the total post-seeding time period for chronology A and 75% for chronology D (Figure 1.3). Despite this, the vg/mL yield for AAV2/8 chronology A is markedly higher than D (Figure 2D). For AAV2, chronologies A and D vg/mL yields are broadly the same (Figure 2C).

Influence of transfection chronology on production of transducing units

We next used standard bench-scale transduction and flow cytometry procedures to quantify the level of GFP payload genome transfer to HEK293T cells (Figure 3), achieved by the cell lysate material tested in Figure 2. We also determined the fluorescence of untreated target cells and plotted this as a notional TU/mL level to represent the LOQ for the procedure. We interpreted TU/mL levels from transduction experiments that were at or below the LOQ as an abolition of infectiousness.

In line with observations reported by Ellis et al.,[43] the AAV2 Bmk transfection resulted in crude lysate with a markedly higher TU/mL yield than AAV2/8 (Figure 3). AAV2 chronologies A, B, D, and E yielded TU/mL levels at or below LOQ (Figure 3A), while all AAV2/8 chronologies exceeded LOQ (Figure 3B). Comparing the vg/mL data of Figure 2 and the TU/mL data of Figure 3, we concluded that associations between capsids and payload genomes were sufficient for nuclease protection but insufficient for infection competence. This may be an indication of the degree to which capsids can adopt a native, infection-competent conformation.[44,45]

For AAV2/8, the relative TU/mL yields of chronologies A–G (Figures 3B and 3D) match the pattern of vg/mL yields (Figures 2B

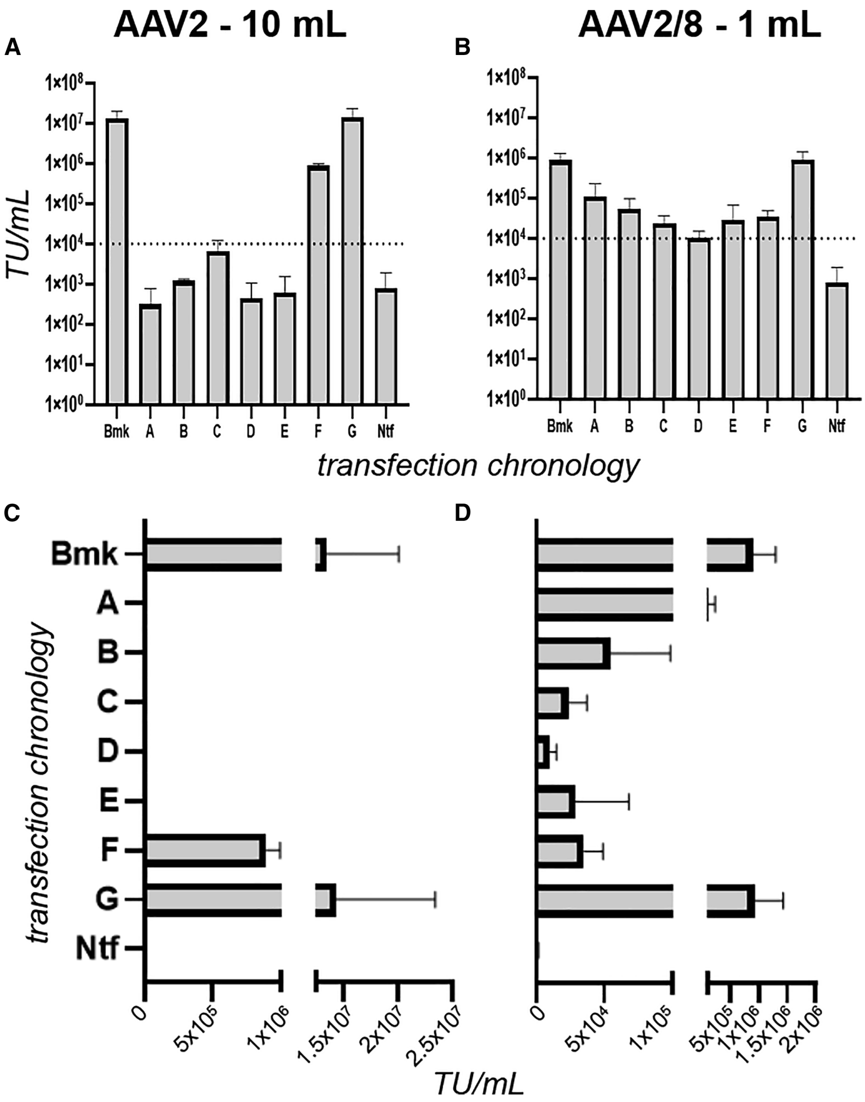

Figure 3. AAV2 and AAV2/8 transducing unit yield performance as a function of transfection chronologically

Volumetric transducing unit titers (TU/mL) were determined by flow cytometry of target cells, to detect GFP expression, for AAV2 and AAV2/8 crude lysates arising from the Figure 1.4 transfection chronologies, at 10 mL and 1 mL scales, respectively. Plots (A) and (C) show AAV2 results on log and linear scales, respectively, with the equivalent for plots (B) and (D) for AAV2/8. Crude lysate from non-transfected cells (Ntf) was incubated with target cells and the resulting fluorescence signal interpreted as the LOQ for the assay. The dotted lines and plots in (A) and (B) highlights the lowest yield achieved by any transfection chronology. Error bars represent standard deviation of two to four biological replicates.

and 2D), with chronology D the lowest in both cases. However, for AAV2 this is not the case, with the vg/mL yield pattern for chronologies A–E being inverted for TU/mL, with chronology C going from lowest (Figures 2A and 2C) to highest (Figure 3A).

Chronology F is the only transfection chronology which, for both AAV2 and AAV2/8, completely omits a plasmid from a transfection time point and yields both measurable vg/mL and TU/mL. As such, we selected chronology F, with Bmk as comparator, for analysis by TEM (Figure 4). TEM images revealed that chronology F was able to yield viral particles of comparable gross morphology to the Bmk chronology, for AAV2 and AAV2/8. A comprehensive structural investigation, beyond the scope of this current investigation, will be needed to achieve more conclusive insights. However, we can conclude from these images that, for both AAV2 and AAV2/8, the reduction in TU/mL, from Bmk chronology to chronology F, was not due to total loss of formation of particles with expected gross morphology.

We next wished to determine if the yield effects of chronology F were an artifact of unexpected toxicity or extreme metabolic burden on host cells. To do this, we monitored the total number and viability of transfected cells over the 72 h post-T1 for chronology F and the Bmk and G chronologies as comparators (Figure S1). No major differences between the chronologies were observed in terms of viable cell number and viability. This was consistent with observations reported by Cervera et al.[34] and Riedl et al.[35] that multiple transfections of a given batch of HEK293 cells did not negatively impact their growth or viability relative to a single transfection procedure.

Influence of transfection chronology on AAV2 capsid yield as determined by ELISA

To determine the level of intact capsid formation for AAV2 transfection chronologies, we used the "AAV2 Titer Capsid ELISA Kit" (Genscript, Oxford, UK, L00942), which, according to the company, recognizes "intact capsids." To confirm that the ELISA kit detection was dependent on epitope conformation, we used purified AAV2 standards and regular cell lysate samples that had been heated at 75°C for 10 min prior to analysis (Figure S2). The heating step resulted in ELISA signal loss, which was consistent with loss of conformational epitope.

Comparison of Figure 5A with Figure 3C shows that the relative pattern of AAV2 capsid/mL yield matched the pattern of TU/mL yield, with respect to transfection chronologies. We interpreted this as evidence that, for AAV2, the ability to achieve the conformational epitope recognized by the ELISA kit antibodies (Figure 5A) correlated with infectiousness (Figure 3C) but did not correlate with the ability to protect viral genomes from nuclease activity (Figures 2A and 2C). Furthermore, the formation of capsids capable

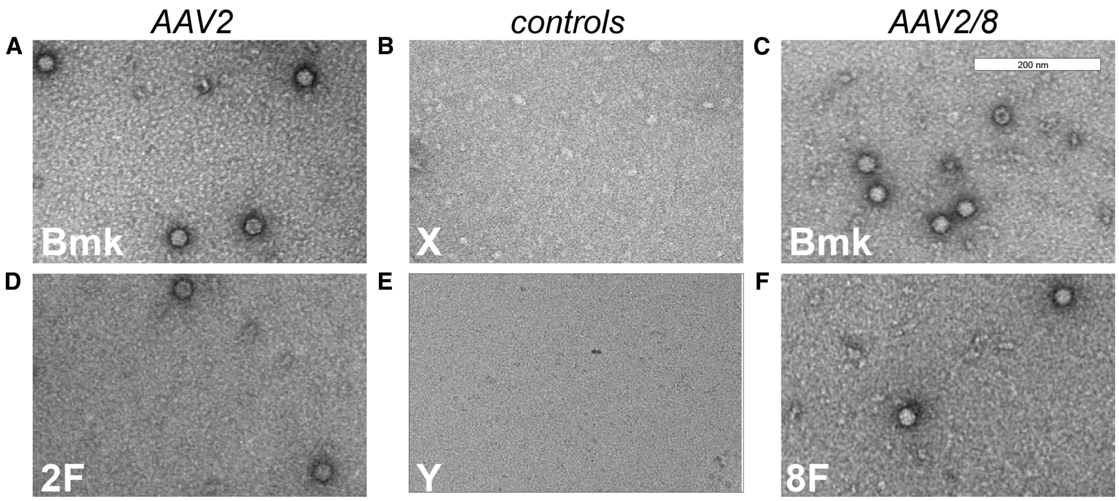

Figure 4. Transfection chronology F produces particles of standard morphology for AAV2 and AAV2/8

Transmission electron microscopy (TEM) analysis of AAV2 and AAV2/8 viral particles. Representative TEM micrographs showing AAV2 (A and D) and AAV2/8 (C and F) particles. Benchmark (Bmk) preparations of both serotypes (A and C) show characteristic ∼28 nm spherical capsids. AAV2 produced using transfection chronology F (D) (labeled "2F") yielded intact particles similar to benchmark. AAV2/8 produced using transfection chronology F (F, labeled "8F") also demonstrated morphology consistent with the benchmark preparation. (B) (labeled "X") is the HEK293F cell lysate that had undergone the same production and sample preparation process as Bmk but without the addition of plasmids and (E) (labeled "Y") is PBS. No particle structures were observed for either. Scale bars represent 200 nm and is applicable to all panels.

of protecting genomes from nuclease activity is less sensitive to transfection chronology than the formation of infectious capsids.

Evidence of AAV2 capsid filling based on ELISA and qPCR data

ELISA and qPCR data can provide a useful indication of the level of correct capsid filling that has been achieved by a given AAV production procedure.[46] A simple approach is to divide vg/mL by capsids/mL (Figure 5B). For AAV2, this approach was only possible for the chronologies (Bmk, F, and G) that yielded measurable capsids/mL levels by ELISA (Figure 5A). For chronologies A–E, although absorbance values were higher than the blank samples (see materials and methods), they were still below the ELISA kit's LOQ. Bmk and chronology G both yielded similar levels of capsid filling, with chronology F resulting in an average 7.5-fold increase in filling. This observation arguably supports our hypothesis that the presence of Helper and Payload plasmid at T1 (Figures 1.3 and 1.4) may "prime" host cells for the subsequent onset of capsid and payload genome production that commences from T2 onward (the latter 54% of the post-seeding period).

Inference of AAV2 capsid filling using a novel, ELISA-free method

To further assess the capsid filling level in AAV2, we sought to trial a novel assay for capsid upload that is ELISA-free (A-CUE), which we would then validate by comparison with the more commonly used method based on qPCR and ELISA data. Step 1 of the A-CUE procedure was to normalize cell lysate samples from each AAV2 transfection chronology with respect to vg/mL, to match total vg content and volume. In step 2, each of these matched samples were then incubated with the same amount of Dynabeads CaptureSelect AAVX

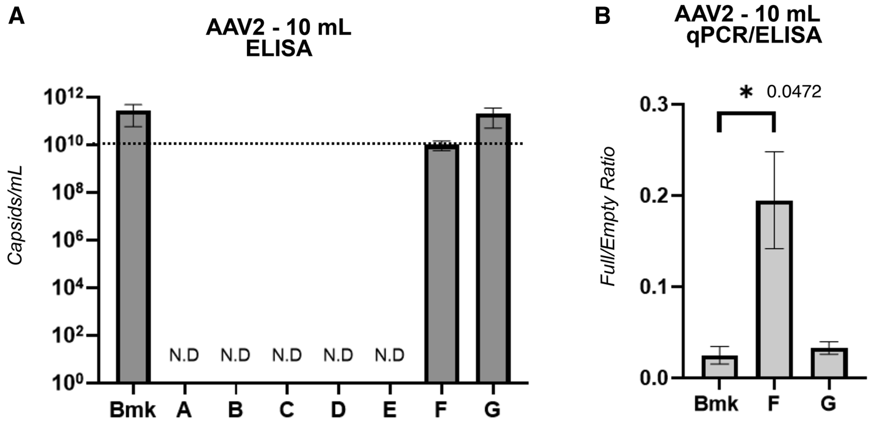

Figure 5. Correctly assembled AAV2 capsid yield and filling as a function of transfection chronology

(A) is a plot of ELISA-based quantification of total correctly assembled capsids (capsids/mL) in crude lysates arising from the Figure 1.4 AAV2 transfection chronologies, performed at 10 mL scale. The dotted line highlights the lowest measurable yield achieved. Non-detectable (N.D.) signal resulted from the indicated chronologies. ELISA data were combined with the AAV2 qPCR data plotted in Figure 2 to calculate inferred levels of capsid filling with payload genomes. The full/empty capsid ratios (qPCR/ELISA) were then plotted in (B), for the chronologies with measurable capsids/mL. Asterisk (*) denotes statistical significance ($p < 0.05$). Error bars represent standard error of the mean from four biological replicates.

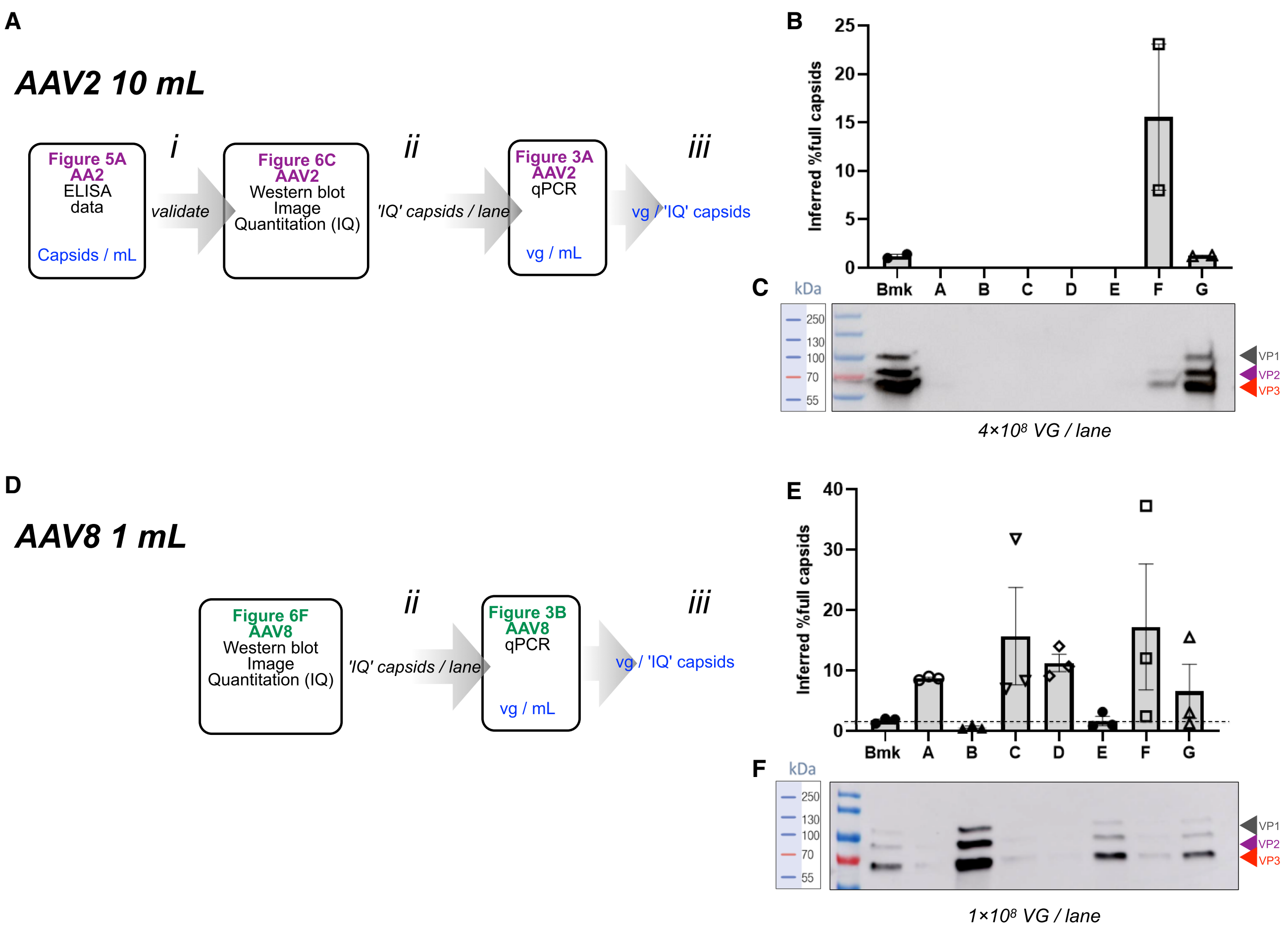

Figure 6. Inferred levels of correct capsid filling using the ELISA-free A-CUE procedure

The novel, "A-CUE", ELISA-free procedure for inferring the level of correct capsid filling with payload genomes is set out in detail in the results section. (A) illustrates a workflow whereby the A-CUE method can be validated against ELISA for AAV2. In (i), comparison of Figure 5A ELISA data and a western blot image arising from the A-CUE procedure in (C) reveals matching patterns of signal as a function of transfection chronology. As such, when western blot images (C) are quantified by antibody binding signal in a given lane (step ii), they can be combined with Figure 2 qPCR data (step iii) to calculate an inferred level of correct capsid filling. (B) plots this inferred correct filling level as a function of transfection chronology, giving a comparable AAV2 filling pattern to Figure 5B. (C) is the western blot image, annotated by red, purple, and black left-pointing triangles, to indicate the VP1, VP2, and VP3 bands. These sizes were inferred by comparison with band migration in the ladder (unlabeled, leftmost lane). (D) is the A-CUE data analysis workflow for AAV2/8, which omits step (i) due to the lack of ELISA data. Steps (ii) and (iii) were then followed, based on the (F) western blot image and the Figure 2 qPCR data. Resulting inferred level of correct capsid filling were plotted in (E). (F) is the western blot, annotated as in (C). For (C) and (F), the gel loading for every transfection chronology was normalized by total number of viral genomes (vgs) present in the bead incubation step. For (B) and (E), error bars represent standard deviation from three biological replicates.

Magnetic Beads (referred to from here as "beads"). These beads have been shown by others to capture AAV2 virus particles in a manner that does not selectively enrich for full or empty capsids.[47–50] As such, they are not expected to introduce any bias into concentration procedures and are understood to capture a representative population of AAV2 particles from a given sample.

After incubation with lysate, for step 3 beads were washed and resuspended in the same volume of protein loading buffer for each sample. No elution step was performed. In step 4, equal volumes of bead solution were analyzed by standard acrylamide gel electrophoresis, with the notional vg count in the bead sample being based on the normalized, known vg counts of lysate that had been incubated with beads in step 1. The gel was processed for western blotting (Figure 6C) using the Progen B1 antibody, which targets a linear epitope, IGTRYLTR, present in the VP1, VP2, and VP3 capsid monomers of multiple AAV serotypes, including AAV2 and AAV2/8.[51]

With respect to transfection chronology, the resulting pattern of western blot antibody binding (Figure 6C) from the A-CUE method approximated the patterns of capsids/mL yield (Figure 5A), as

determined by ELISA, and TU/mL yield (Figures 3A and 3C). The similar patterns of A-CUE data, ELISA data, and infectivity data for AAV2 (Figures 3, 5, and 6) suggested that the A-CUE method could be a viable alternative to ELISA for estimating capsid filling in combination with qPCR data (Figure 6A for workflow). As such, we proceeded to used ImageJ to quantify an antibody binding signal in each western blot lane. We termed these quantification values as "image quantification capsids" (IQ capsids)/lane. We then divided "IQ capsids"/lane by "vg loaded"/lane and plotted this as "inferred percentage full capsids" (Figure 6B). These inferred filling levels arising from the A-CUE procedure closely tracked those determined in Figure 5B by ELISA and qPCR, with both methods indicating that chronologies Bmk and G resulted in similar filling, while chronology F had an approximately 7.5-fold higher level.

Inference of AAV2/8 capsid filling based on the novel "A-CUE" procedure

Given the alignment between the ELISA-and-qPCR method and the A-CUE methods for determining AAV2 capsid filling, we next sought to use A-CUE alone to determine AAV2/8 capsid filling, in the absence of an effective AAV8 ELISA kit. We processed samples to the western blot stage as before (Figure 6F) and divided the resulting "IQ capsids" per lane value with the matched "vg per lane" values to inferred percentage full capsids for each AAV2/8 transfection chronology (Figure 6E). In common with AAV2, the levels of AAV2/8 capsid filling resulting from chronology F were higher than those for the Bmk chronology and chronology G. The Figure 6F western blot image revealed that every AAV2/8 chronology yielded a detectable level of antibody binding, including a VP3 band that is visible for chronology D upon close inspection. By contrast, AVV2 chronologies B, C, and D now show western blot signals, even after close inspection (Figure 6C).

The A-CUE data for AAV2/8 (Figure 6E) suggested that chronologies A, C, D, and G all exceeded the capsid filling level of the Bmk transfection. However, this filling pattern for AAV2/8 did not map onto TU/mL (Figures 3B and 3D) or vg/mL (Figures 2B and 2D) yield performance.

DISCUSSION

With respect to AAV2 process understanding, only transfection chronologies F and G (where both Payload and Helper plasmid were present in the first transfection) yielded both infectious virus and ELISA-positive capsids. All other chronologies abolished either production of infectious capsids or ELISA detection, or both, indicating almost no correctly assembled capsids could be formed in a temporally misaligned AAV2 production process. By contrast, all AAV2/8 transfection chronologies yielded infectious viruses. This difference between AAV2 and AAV2/8 is consistent with AAV capsid serotype-specific differences in optimal scheduling of synthesis and assembly events required for correct capsid assembly, observed by Earley et al.[52]

The data in Figure 6 are consistent with transfection chronology F, resulting in the highest level of filling for both AAV2 and AAV2/8. Compared to a conventional Bmk transfection, chronology F has a 20-h delay in the introduction of RepCap plasmid into host cells that are already theoretically capable of viral genome replication (Figure 1.4). As such, it is possible that chronology F represents a more productive temporal alignment of genome replication, followed by capsid assembly, in agreement with the TMA hypothesis.

The AAV2/8 transfection chronology that yielded the lowest TU/mL average overall was chronology D, where both RepCap and Payload plasmids were present at T1 (Figures 3B and 3D). This was lower than chronology A, which featured only RepCap plasmid for the T1 transfection. One possibility is that the suboptimal peak of capsid synthesis predicted by TMA was further exacerbated by the presence of both the RepCap and Payload plasmids together (chronology D) compared to the presence of RepCap alone (chronology A).

For chronologies B, C, and F, RepCap plasmid is only present for 54% of the post-seeding period, compared to 75% for the Bmk transfection. As such, capsid yields for B, C, and F might be expected to be less than that of Bmk. For AAV2, this is indeed the case, with F having less capsids and both B and C having no detectable capsids, for both ELISA (Figure 5A) and A-CUE data (Figure 6C). For AAV2/8, while C and E did yield less capsid than Bmk, chronology B yielded significantly more capsid signal than Bmk, as determined by A-CUE procedure (Figure 6F). We interpreted this as evidence of divergence between serotypes in terms of optimal viral assembly schedule. Ohba et al. also reported observations consistent with serotype-based differences in viral assembly schedule. When they placed Cap gene expression under inducible control for AAV serotypes 1, 2, 3, 5, 6, 7, 8, 9, and 10, only AAV8 did not show an improvement in capsid filling levels when Cap expression was delayed relative to Rep gene expression.[21]

From a bioprocess perspective, for AAV2 and AAV2/8, chronology G matched but did not exceed the performance of the conventional, single-transfection Bmk procedure in terms of resultant vg/mL, TU/mL, and capsid filling. As such, for non-automated systems, the additional human operator labor required for the second transfection would not be justified. Chronology F achieved a 7.5-fold increase in filling for AAV2 (Figures 5 and 6), with a similar increase in filling hinted at in the A-CUE data arising from AAV2/8 (Figure 6). When considering implementation, the process benefit of chronology F would have to be balanced against the additional operator labor and the reductions in vg/mL and TU/mL yield performance. For both AAV2 and AAV2/8, there may be a sweet spot between chronologies G and F, where the vg/mL and TU/mL could be enhanced by a small dose of RepCap plasmids delivered alongside Helper and payload at the first point, while the capsid filling levels could be improved by a larger second dose of RepCap at the second time point. Further empirical studies may identify this optimized chronology.

In conclusion, our systematic investigation of chronologically distributed transfection revealed several important insights into AAV production dynamics. The most striking finding was the

serotype-specific difference in tolerance to varied transfection schedules, with AAV2/8 demonstrating greater robustness than AAV2 across different chronologies, and it suggests bespoke optimization strategies may be needed for each serotype.[53,54]

The success of chronology F in improving capsid filling efficiency, particularly for AAV2, provides empirical support for the temporal misalignment hypothesis and suggests that strategic scheduling of plasmid delivery can enhance production outcomes. However, the trade-off between improved filling and reduced overall yields highlights the complexity of optimizing AAV production processes. These findings not only advance our understanding of AAV assembly schedules but also provide practical considerations for process development in gene therapy manufacturing. Future studies combining this chronological approach with detailed molecular analyses of intracellular events could further elucidate the mechanisms underlying serotype-specific assembly patterns and guide the development of more efficient production strategies.

MATERIALS AND METHODS

Plasmid identities and propagation

AAV genomic payload for all transfections was encoded by pAAV-GFP (Cell Biolabs, AAV-400). AAV2 RepCap genes were encoded by pRC2 (Cell Biolabs,VPK-422). The plasmid pAAV2/8 (Addgene plasmid #112864), encoding AAV2 *Rep* and AAV8 *Cap* genes, was used for AAV2/8 production, and the Helper plasmid for all transfections was pAdDeltaF6 (Addgene plasmid #112867), both kind gifts from James M. Wilson, University of Pennsylvania, USA. Standard molecular biology techniques were used for all plasmid propagation, purification, and analytical procedures.

Mammalian cell cultivation

FreeStyle HEK293-F cells (Life Technologies Limited, Paisley, UK, catalog number R79007) were cultivated in FreeStyle 293 Expression Medium (Life Technologies, cat. no. 12338018) at 37°C with 5% CO_2 and shaken at 135 rpm inside a humidified incubator (PHC Europe B.V., Loughborough, UK, model MCO-170AICD-PE). Adherent HEK293T cells (cat. no. CRL-3216) from American type culture collection (ATCC) were cultivated using Dulbecco's modified Eagle's medium (DMEM) with 10% v/v heat-inactivated fetal bovine serum (FBS), both purchased from Gibco, UK. Cells were kept at 37°C, 5% CO_2 also in a PHC Europe, model MCO-170AICD-PE, humidified incubator.

Transient transfection

All transfection chronologies are depicted in Table 1 and were performed as follows. Twenty-four hours prior to transfection, HEK293-F host cells were seeded at 5×10^5 cells/mL in FreeStyle 293 Expression Medium, either in six-well plates (Sarstedt, Germany, 83.3920.500), with a total volume of 1 mL per well, or in 125 mL shake flasks (Corning 431143) with a total volume of 10 mL per flask. For the Bmk transfection, Helper, RepCap, and Payload plasmid solutions were combined in a 1:1:1 molar ratio, to a final overall plasmid concentration of 1.5 μg/mL, and then diluted with Opti-MEM (Thermo Fisher Scientific, Lough borough, UK, 12559099) to a volume equivalent to 5% of the total cell culture volume. A total of 1.5 μL PEIpro (Sartorius Stedim, Epsom, UK, 101000017) per 1.5 μg of plasmid was diluted with Opti-MEM to the same total volume as the plasmid DNA solution. PEI and DNA solutions were then combined and incubated at room temperature (RT) for 15 min for complex formation. This PEI-DNA solution was then added dropwise to suspension cell cultures.

Transfections with chronologies A–G were performed using the same mass of each plasmid as used in the Bmk transfection procedure, but split across two transfections, performed at 24 and 44 h after seeding of host cells. Each transfection featured either one or two plasmids, with PEIpro volume scaled down on the basis of a 1:1 PEI volume-to-mass of plasmid ratio (μL:μg). PEI and DNA solutions were each made up to a total Opti-MEM volume proportional to the plasmid volume, with specific volumes provided in Table 2. These solutions were combined for complexing and added to cells as in the Bmk procedure.

AAV harvest

For all transfection procedures, a volume of the Gibco AAV-MAX Lysis Buffer (Thermo Fisher Scientific, cat. no. 17331899) equivalent to 10% of total cell culture volume was added to host cells 96 h post-seeding, and the cell culture vessel returned to the incubator for 2 h under standard cultivation conditions. The total volume of cell culture plus lysis buffer was then transferred to falcon tubes and centrifuged at 4,500 rpm for 30 min at 4°C (Eppendorf, UK, 5910R). The supernatant was gently removed, taking care not to disrupt a small pellet, aliquoted and stored at −80°C prior to further manipulations.

Real-time quantitative PCR

AAV payload viral genomes per mL (vg/mL) in crude lysate were determined by qPCR. A 5 μL crude lysate sample, 35 μL of molecular biology grade water (Corning, UK, 46-000-CM), 5 μL of 10× DNAse I reaction buffer (New England Biolabs, Hitchin, UK, M0303S), and 5 μL of 2 U/μL DNase I (New England Biolabs, Hitchin, UK, M0303S) were added together followed by a 30-min incubation at 37°C, in order to degrade all DNA not sequestered within AAV capsids. Samples were then incubated at 95°C for 10 min to denature capsids. Primers of sequence GTCCGCCCTGAGCAAAGA and TCCAGCAGGACCATGTGATC[55] were then added to PCR-amplify the segment of the AAV genome encoding the green fluorescent protein (GFP) open reading frame (ORF). Dilutions of the linearized pAAVGFP plasmid of known purity and concentration were used to establish a standard curve of cycle threshold (Ct) determinations. Serially diluted samples were mixed with SsoAdvanced Universal SYBR Green Supermix (Bio-Rad, Watford, UK, 1725271) and loaded onto the CFx Connect device (Bio-Rad, UK), set for a 3-min incubation at 98°C for polymerase activation and DNA denaturation, followed by 39 cycles of amplifications. Each round of amplification consisted of a 15-s, 98°C denaturation step and a 30-s, 60°C annealing/extension step. Melt curve analysis was then performed in an automated manner by the CFx Connect device software.

Enzyme-linked immunosorbent assay

To quantify the number of intact AAV2 particles, the AAV2 Titer Capsid ELISA Kit (Genscript, Oxford, UK, L00942) was used according to the manufacturer's instructions. Briefly, crude lysate samples from AAV2 production and the AAV2 controls provided with the kit were serially diluted and loaded onto a 96-well plate coated with the capture antibody. This was followed by incubation with Biotin anti-AAV2 antibody and the streptavidin-horseradish peroxidase conjugate (streptavidin-HRP), with washing steps between incubations. The 3,3′,5,5′-tetramethylbenzidine solution (TMB Solution) was then added, followed by stop solution. The intensity of the color in each well was measured at 450 and 650 nm using the CLARIOstar plate reader (BMG LABTECH, Aylesbury, UK). To eliminate the background signal from turbidity, the absorbance at 650 nm was subtracted from the absorbance at 450 nm of the same well. A standard curve was established using a four-parameter logistic (4-PL) model, with the AAV2 control concentration (capsids/mL) plotted on the x axis and the corresponding mean absorbance value on the y axis in GraphPad Prism 9.4.1. LOQ was set up according to the CLSI method guideline EP17-A2 by the manufacturer. It was defined as the actual concentration at which the analyte is reliably detected and the uncertainty of the observed test result is less than or equal to the goal set by the laboratory (10% coefficient of variation).

Viral transduction and flow cytometry

Fifty microliters of a solution of adherent HEK293T cells was seeded as target cells at 1×10^6 cells/mL in 10% v/v FBS DMEM into each well of a 96-well plate (Thermo Fisher Scientific, Loughborough, UK, 10212811) to give a total of 5×10^4 cells/well. One hour post-seeding, serially diluted samples of crude lysate were added to each well to a total volume of 12 μL. Twenty-four hours after lysate sample addition, 100 μL of 10% v/v FBS DMEM was added to each well. Seventy-two hours after lysate sample addition, all liquid was removed from each well, cells were harvested by standard trypsinization procedure (Gibco, 25200056), and then fixed by incubation with a solution (Thermo Fisher Scientific, cat. no. 15424389) of 4% v/v paraformaldehyde solution in phosphate-buffered saline (PBS) for 10 min. These fixed cell solutions were centrifuged at 1,000 rpm (Eppendorf, 5910R), supernatants removed, and cell pellets resuspended in PBS in round bottom 96-well plates (Thermo Fisher Scientific, 268200) prior to flow cytometry.

To quantify fluorescence arising from viral transfer of the GFP-encoding AAV payload genome, an excitation wavelength of 488 nm was used for the LSRFortessa Cell Analyzer Flow Cytometer (BD Biosciences, UK). A minimum of 10,000 cell measurement events were recorded per sample. Data gathered from the flow cytometry experiments were analyzed using FlowJo v.10.8.1 (BD Biosciences, UK) software. To count the number of live GFP-positive cells, forward and side scatter gating analyses were applied to isolate live cells and forward scatter height and forward scatter area were applied to isolate singlet cells. The percentage of GFP-positive cells was measured and normalized against non-transduced cells. The number of transducing units/mL was calculated using the following formula:

$$\text{TU/mL} = \{(\text{\% GFP-positive cells})^*(\text{no. cells at transduction})]/\text{vector input volume}\}^*\text{dilution factor} \quad \text{(Equation 1)}$$

Bead capture and electrophoresis of AAV particles

As part of the A-CUE procedure, the vg/mL levels in AAV2 and AAV2/8 cell lysates were used to inform sample dilution with PBS (Thermo Fisher Scientific, 12549079) to achieve the same vg concentration and volume (1.8 mL) for all samples for a given serotype (4×10^8 vg/sample for AAV2 and 1×10^8 vg/sample for AAV2/8). These vg-matched samples were then mixed with the same volume of prewashed Dynabeads CaptureSelect AAVX Magnetic Bead solution. The lysate/bead slurry was then placed on a tube rotator (Cole-Parmer,TR-200) at 20 rpm for 30 min to allow binding to occur. Sample tubes were then placed in an Invitrogen DynaMag-2 Magnet (Thermo Fisher Scientific, 10723874) to immobilize the beads. While beads were immobilized, lysate was removed and two PBS washes performed, each with typically 500 μL PBS. Washed beads were then resuspended in protein loading buffer, which is composed of 30 μL 1× Gibco AAV-MAX Lysis buffer (Thermo Fisher Scientific) plus 9 μL of 4X Laemmli buffer (Bio-Rad, Watford, UK, 1610747) and 1 μL of 2-mercaptoethanol (Bio-Rad, Watford, UK, 1610710). This sample was then incubated at 95°C for 10 min in a ThermoMixer C (Eppendorf, UK, 15158953) with the purpose of denaturing capsid proteins such that bead-bound particles would disassemble into soluble constituent proteins. Samples were then placed in the Invitrogen DynaMag-2 Magnet (Thermo Fisher Scientific, 10723874), and the supernatant was removed and loaded onto a 10% Mini-PROTEAN TGX Protein Gel for electrophoresis at 160 volts for 40 min before being processed for western blotting.

Western blotting

PROTEAN TGX Protein Gel was transferred to nitrocellulose membranes using the Trans-Blot Turbo Mini 0.2 μm Nitrocellulose Transfer Pack (Bio-Rad, cat. no. 1704158) and the Trans-Blot Turbo Transfer System (model 1704150). Membranes were blocked with EveryBlot Blocking Buffer (Bio-Rad, cat. no. 12010020) for 5 min, and five 5-minute washes with Tris-buffered saline and 0.05% v/v Tween 20 (Bio-Rad, 1706435 and Merck Life Science, Gillingham, UK, P9416) were performed after each antibody incubation step. The anti-AAV VP1/VP2/VP3 mouse monoclonal (PROGEN, cat. no. 690058) was added first, at 1/250 dilution before incubation at RT for 1.5 h with rocking. Rabbit Anti-Mouse IgG H&L (HRP) antibody (Abcam, Cambridge, UK, AB6728-1001) was then added at 1/2,000 dilution prior to 1-h RT incubation. Bound antibodies were detected using Clarity MAX ECL substrate (Bio-Rad, cat. no. 1705062) and the luminescent image analyzer (Amersham Imager 600, Cytiva).

Densitometric analysis of western blot band intensity was performed using ImageJ (National Institutes of Health, USA) without any manipulation of image brightness or contrast. The acquired images were black/white inverted in ImageJ prior to analysis. The "rectangular selection" tool was used to draw a region of interest (ROI) encompassing each VP protein band and a blank area. The same ROI

size was applied to all VP bands to ensure consistency. The intensity of each band in a gel image was normalized by subtracting the intensity of a blank area of the ROI size within the same gel.

Transmission electron microscopy

To prepare samples for transmission electron microscopy (TEM), the productions were linearly scaled up to 50 mL for AAV2 and 12.5 mL for AAV8 in 250 mL and 125 mL Erlenmeyer shake flasks (Coring, 431143). After the 72-h production period, the cell culture was centrifuged at 2,000 rpm for 15 min, then resuspended in PBS to 4% of the original cell culture volume. A volume of Gibco AAV-MAX Lysis buffer (Thermo Fisher Scientific, 17331899) equivalent to 10% of the cell suspension was added, followed by 2-h incubation under standard cultivation conditions. The cell suspension was subjected to three cycles of freezing and thawing process between a dry ice/70% ethanol bath and a 37°C water bath; 0.8 μL (200 units) of Benzonase nuclease (Merck KGaA, Darmstadt, Germany, E1014) was added to each sample and incubated at 37°C for 1 h. The cell lysate was then centrifuged at 12,000 rpm for 10 min and the supernatant removed and stored at 4°C prior to purification. Manufacturer's instructions for the Dynabeads CaptureSelect AAVX Magnetic Beads (Life Technologies Limited, Paisley, UK, 2853522001) system were then followed to purify viral particles from crude lysate. Briefly, beads were incubated with sample for 30 min before two washes and a final elution step in 60 μL of elution buffer and 6 μL of neutralization buffer.

Formvar/Carbon films on 200-mesh copper grids (TAAB, Berkshire, UK, F077/100) were glow-discharged using the PELCO easiGlow machine. Three microliters of purified samples were loaded onto the grid and incubated for 2 min at RT. The excess sample was removed. Three microliters of 1% v/v uranyl acetate was loaded onto the grid and incubated for 40 s at RT. The samples were dried for around 20 min prior to imaging with the TEM (T12 Tecnai Spirit BioTwin, FEI).

DATA AND CODE AVAILABILITY

Raw data supporting the findings of this study are available from the corresponding author on request.

ACKNOWLEDGMENTS

We gratefully acknowledge the support of the UK Engineering and Physical Sciences Research Council (EPSRC) via grant number EP/X025446/1.

AUTHOR CONTRIBUTIONS

Conceptualization, methodology, investigation, visualization, and supervision, Q.C., R.W., D.N.N., and C.H.L.; writing—original draft and writing—review & editing, Q.C., R.W., and D.N.N.

DECLARATION OF INTERESTS

The authors declare no competing interests.

SUPPLEMENTAL INFORMATION

Supplemental information can be found online at https://doi.org/10.1016/j.omtm.2025.101610.

REFERENCES

1. Wang, D., Tai, P.W.L., and Gao, G. (2019). Adeno-associated virus vector as a platform for gene therapy delivery. Nat. Rev. Drug Discov. *18*, 358–378.
2. Planul, A., and Dalkara, D. (2017). Vectors and Gene Delivery to the Retina. Annu. Rev. Vis. Sci. *3*, 121–140.
3. Wang, L., Wang, H., Bell, P., McMenamin, D., and Wilson, J.M. (2012). Hepatic Gene Transfer in Neonatal Mice by Adeno-Associated Virus Serotype 8 Vector. Hum. Gene Ther. *23*, 533–539.
4. Wright, J.F. (2008). Manufacturing and characterizing AAV-based vectors for use in clinical studies. Gene Ther. *15*, 840–848.
5. Tse, L.V., Moller-Tank, S., and Asokan, A. (2015). Strategies to circumvent humoral immunity to adeno-associated viral vectors. Expert Opin. Biol. Ther. *15*, 845–855.
6. Xiao, X., Li, J., and Samulski, R.J. (1998). Production of High-Titer Recombinant Adeno-Associated Virus Vectors in the Absence of Helper Adenovirus. J. Virol. *72*, 2224–2232.
7. Large, E.E., Silveria, M.A., Zane, G.M., Weerakoon, O., and Chapman, M.S. (2021). Adeno-associated virus (AAV) gene delivery: dissecting molecular interactions upon cell entry. Viruses *13*, 1336.
8. Samulski, R.J., Srivastava, A., Berns, K.I., and Muzyczka, N. (1983). Rescue of adeno-associated virus from recombinant plasmids: Gene correction within the terminal repeats of AAV. Cell *33*, 135–143.
9. Samulski, R.J., Chang, L.S., and Shenk, T. (1989). Helper-free stocks of recombinant adeno-associated viruses: normal integration does not require viral gene expression. J. Virol. *63*, 3822–3828.
10. King, J.A., Dubielzig, R., Grimm, D., and Kleinschmidt, J.A. (2001). DNA helicase-mediated packaging of adeno-associated virus type 2 genomes into preformed capsids. EMBO J. *20*, 3282–3291.
11. Doshi, J., Couto, E., Staiti, J., Vandenberghe, L.H., and Zabaleta, N. (2024). E2A, VA RNA I, and L4-22k adenoviral helper genes are sufficient for AAV production in HEK293 cells. Mol. Ther. Methods Clin. Dev. *32*, 101376.
12. Ward, P., Clément, N., and Linden, R.M. (2007). cis Effects in Adeno-Associated Virus Type 2 Replication. J. Virol. *81*, 9976–9989.
13. Grimm, D., Kern, A., Pawlita, M., Ferrari, F., Samulski, R., and Kleinschmidt, J. (1999). Titration of AAV-2 particles via a novel capsid ELISA: packaging of genomes can limit production of recombinant AAV-2. Gene Ther. *6*, 1322–1330.
14. Zeltner, N., Kohlbrenner, E., Clément, N., Weber, T., and Linden, R.M. (2010). Near-perfect infectivity of wild-type AAV as benchmark for infectivity of recombinant AAV vectors. Gene Ther. *17*, 872–879.
15. Cao, L., During, M., and Xiao, W. (2002). Replication competent helper functions for recombinant AAV vector generation. Gene Ther. *9*, 1199–1206.
16. Liu, H., Liu, N., Zhou, C., Du, A., Kapadia, M., Tai, P.W.L., Barton, E., Gao, G., and Wang, D. (2025). High-purity AAV vector production utilizing recombination-dependent minicircle formation and genetic coupling. EMBO Mol. Med. *17*, 1475–1494.
17. Nguyen, T.N.T., Sha, S., Hong, M.S., Maloney, A.J., Barone, P.W., Neufeld, C., Wolfrum, j., Springs, S.L., Sinskey, A.J., and Braatz, R.D. (2021). Mechanistic model for production of recombinant adeno-associated virus via triple transfection of HEK293 cells. Mol. Ther. Methods Clin. Dev. *21*, 642–655.
18. Lee, Z., Lu, M., Irfanullah, E., Soukup, M., and Hu, W.-S. (2022). Construction of an rAAV Producer Cell Line through Synthetic Biology. ACS Synth. Biol. *11*, 3285–3295.
19. Lu, M., Lee, Z., Lin, Y.C., Irfanullah, I., Cai, W., and Hu, W.S. (2024). Enhancing the production of recombinant adeno-associated virus in synthetic cell lines through systematic characterization. Biotechnol. Bioeng. *121*, 341–354.
20. Lu, M., Lin, Y.C., Kuo, H.J., Cai, W., Ye, Q., Zhao, L., and Hu, W.S. (2024). Tuning capsid formation dynamics in recombinant adeno-associated virus producing synthetic cell lines to enhance full particle productivity. Biotechnol. J. *19*, 2400051.
21. Ohba, K., Sehara, Y., Enoki, T., Mineno, J., Ozawa, K., and Mizukami, H. (2023). Adeno-associated virus vector system controlling capsid expression improves viral quantity and quality. iScience *26*, 106487.

22. Srinivasan, P., Canova, C.T., Sha, S., Nguyen, T.N.T., Joseph, J., Sangerman, J., Maloney, A.J., Katsikis, G., Ou, R.W., Hong, M.S., et al. (2024). Multidose transient transfection of human embryonic kidney 293 cells modulates recombinant adeno-associated virus2/5 Rep protein expression and influences the enrichment fraction of filled capsids. Biotechnol. Bioeng. *121*, 3694–3714.

23. Lechardeur, D., Sohn, K.J., Haardt, M., Joshi, P.B., Monck, M., Graham, R.W., Beatty, B., Squire, J., O'Brodovich, H., and Lukacs, G.L. (1999). Metabolic instability of plasmid DNA in the cytosol: a potential barrier to gene transfer. Gene Ther. *6*, 482–497.

24. Wang, X., Le, N., Denoth-Lippuner, A., Barral, Y., and Kroschewski, R. (2016). Asymmetric partitioning of transfected DNA during mammalian cell division. Proc. Natl. Acad. Sci. USA *113*, 7177–7182.

25. Pistek, M., Andorfer, P., Grabherr, R., Kraus, B., and Hernandez Bort, J.A. (2024). Factors affecting rAAV titers during triple-plasmid transient transfection in HEK-293 cells. Biotechnol. Lett. *46*, 945–959.

26. Suleman, S., Fawaz, S., Roberts, T., Ellison, S., Bigger, B., and Themis, M. (2024). Optimised protocols to generate high titre lentiviral vectors using a novel transfection agent enabling extended HEK293T culture following transient transfection and suspension culture. J. Virol. Methods *325*, 114884.

27. Puente-Massaguer, E., Strobl, F., Grabherr, R., Striedner, G., Lecina, M., and Gòdia, F. (2020). PEI-mediated transient transfection of high five cells at bioreactor scale for HIV-1 VLP production. Nanomaterials *10*, 1580.

28. Li, R. (2015). Transient transfection of CHO cells using linear polyethylenimine is a simple and effective means of producing rainbow trout recombinant IFN-γ protein. Cytotechnology *67*, 987–993.

29. Bennett, A., Mietzsch, M., and Agbandje-McKenna, M. (2017). Understanding capsid assembly and genome packaging for adeno-associated viruses. Future Virol. *12*, 283–297.

30. Taylor, N.K., Guggenbiller, M.J., Mistry, P.P., King, O.D., and Harper, S.Q. (2024). A self-complementary AAV proviral plasmid that reduces cross-packaging and ITR promoter activity in AAV vector preparations. Mol. Ther. Methods Clin. Dev. *32*, 101295.

31. Jen, H.-I., Wilkinson, P., Lu, X., and Zhang, W. (2025). Residual DNA Impurities in AAV Vectors–Nature and Transcription. Mol. Ther. Methods Clin. Dev. *33*, 101503.

32. Halbert, C.L., Metzger, M.J., Lam, S.-L., and Miller, A.D. (2011). Capsid-expressing DNA in AAV vectors and its elimination by use of an oversize capsid gene for vector production. Gene Ther. *18*, 411–417.

33. Hauck, B., Murphy, S.L., Smith, P.H., Qu, G., Liu, X., Zelenaia, O., Mingozzi, F., Sommer, J.M., High, K.A., and Wright, J.F. (2009). Undetectable transcription of cap in a clinical AAV vector: implications for preformed capsid in immune responses. Mol. Ther. *17*, 144–152.

34. Cervera, L., Gutiérrez-Granados, S., Berrow, N.S., Segura, M.M., and Gòdia, F. (2015). Extended gene expression by medium exchange and repeated transient transfection for recombinant protein production enhancement. Biotechnol. Bioeng. *112*, 934–946.

35. Riedl, S.A.B., Jérôme, V., and Freitag, R. (2022). Repeated transient transfection: an alternative for the recombinant production of difficult-to-express proteins like BMP2. Processes *10*, 1064.

36. Zhang, J., Frabutt, D.A., Chrzanowski, M., Li, N., Miller, L.M., Tian, J., Mulcrone, P. L., Lam, A.K., Draper, B.E., Jarrold, M.F., et al. (2024). A novel class of self-complementary AAV vectors with multiple advantages based on cceAAV lacking mutant ITR. Mol. Ther. Methods Clin. Dev. *32*, 101206.

37. Nash, K., Chen, W., and Muzyczka, N. (2008). Complete in vitro reconstitution of adeno-associated virus DNA replication requires the minichromosome maintenance complex proteins. J. Virol. *82*, 1458–1464.

38. van Lieshout, L., Ota, S., Adusei, A., Wiberg, E., Costa-Grant, K., Lata, D., Dollive, S., Stanvick, M., Iwuchukwu, I., Golebiowski, D., and Yin, J. (2024). An Improved Helper Plasmid Containing Deletions Within the E4 and E2a Genes Results in Increased Adeno-Associated Virus Productivity. Hum. Gene Ther. *35*, 767–776.

39. Martinez-Fernandez de la Camara, C., McClements, M.E., and MacLaren, R.E. (2021). Accurate Quantification of AAV Vector Genomes by Quantitative PCR. Genes *12*, 601.

40. Bucher, K., Rodríguez-Bocanegra, E., Wissinger, B., Strasser, T., Clark, S.J., Birkenfeld, A.L., Siegel-Axel, D., and Fischer, M.D. (2023). Extra-viral DNA in adeno-associated viral vector preparations induces TLR9-dependent innate immune responses in human plasmacytoid dendritic cells. Sci. Rep. *13*, 1890.

41. Chung, C.-H., Murphy, C.M., Wingate, V.P., Pavlicek, J.W., Nakashima, R., Wei, W., McCarty, D., Rabinowitz, J., and Barton, E. (2023). Production of rAAV by plasmid transfection induces antiviral and inflammatory responses in suspension HEK293 cells. Mol. Ther. Methods Clin. Dev. *28*, 272–283.

42. Mouw, M.B., and Pintel, D.J. (2000). Adeno-Associated Virus RNAs Appear in a Temporal Order and Their Splicing Is Stimulated during Coinfection with Adenovirus. J. Virol. *74*, 9878–9888.

43. Ellis, B.L., Hirsch, M.L., Barker, J.C., Connelly, J.P., Steininger, R.J., and Porteus, M. H. (2013). A survey of ex vivo/in vitro transduction efficiency of mammalian primary cells and cell lines with Nine natural adeno-associated virus (AAV1-9) and one engineered adeno-associated virus serotype. Virol. J. *10*, 74.

44. François, A., Bouzelha, M., Lecomte, E., Broucque, F., Penaud-Budloo, M., Adjali, O., Moullier, P., Blouin, V., and Ayuso, E. (2018). Accurate Titration of Infectious AAV Particles Requires Measurement of Biologically Active Vector Genomes and Suitable Controls. Mol. Ther. Methods Clin. Dev. *10*, 223–236.

45. Riyad, J.M., and Weber, T. (2021). Intracellular trafficking of adeno-associated virus (AAV) vectors: challenges and future directions. Gene Ther. *28*, 683–696.

46. Kontogiannis, T., Braybrook, J., McElroy, C., Foy, C., Whale, A.S., Quaglia, M., and Smales, C.M. (2024). Characterization of AAV vectors: A review of analytical techniques and critical quality attributes. Mol. Ther. Methods Clin. Dev. *32*, 101309.

47. Mietzsch, M., Kamat, M., Basso, K., Chipman, P., Huiskonen, J.T., and McKenna, R. (2024). Structural characterization and epitope mapping of the AAVX affinity purification ligand. Mol. Ther. Methods Clin. Dev. *32*, 101377.

48. Terova, O., Soltys, S., Hermans, P., De Rooij, J., and Detmers, F. (2018). Overcoming downstream purification challenges for viral vector manufacturing: enabling advancement of gene therapies in the clinic. Cell Gene Ther. Insights *4*, 101–111.

49. Leibiger, T.M., Remmler, L.A., Green, E.A., and Lee, K.H. (2024). Biolayer interferometry for adeno-associated virus capsid titer measurement and applications to upstream and downstream process development. Mol. Ther. Methods Clin. Dev. *32*, 101306.

50. Florea, M., Nicolaou, F., Pacouret, S., Zinn, E.M., Sanmiguel, J., Andres-Mateos, E., Unzu, C., Wagers, A.J., and Vandenberghe, L.H. (2023). High-efficiency purification of divergent AAV serotypes using AAVX affinity chromatography. Mol. Ther. Methods Clin. Dev. *28*, 146–159.

51. Wobus, C.E., Hügle-Dörr, B., Girod, A., Petersen, G., Hallek, M., and Kleinschmidt, J.A. (2000). Monoclonal antibodies against the adeno-associated virus type 2 (AAV-2) capsid: epitope mapping and identification of capsid domains involved in AAV-2–cell interaction and neutralization of AAV-2 infection. J. Virol. *74*, 9281–9293.

52. Earley, L.F., Powers, J.M., Adachi, K., Baumgart, J.T., Meyer, N.L., Xie, Q., Chapman, M.S., and Nakai, H. (2017). Adeno-associated virus (AAV) assembly-activating protein is not an essential requirement for capsid assembly of AAV serotypes 4, 5, and 11. J. Virol. *91*, e01980-16. https://doi.org/10.1128/jvi.01980-16.

53. Grimm, D. (2002). Production methods for gene transfer vectors based on adeno-associated virus serotypes. Methods *28*, 146–157.

54. Khaparde, A., Patra, R., Roy, S., Babu G R, S., and Ghosh, A. (2025). Improving AAV Production Yield and Quality for Different Serotypes Using Distinct Processing Methods. ACS Omega *10*, 22657–22670.

55. Wang, Y., Menon, N., Shen, S., Feschenko, M., and Bergelson, S. (2020). A qPCR Method for AAV Genome Titer with ddPCR-Level of Accuracy and Precision. Mol. Ther. Methods Clin. Dev. *19*, 341–346.

OMTM, Volume *33*

Supplemental information

Chronologically distributed transfection improves AAV2 and AAV2/8 capsid filling and reveals assembly schedule divergence

Qiantong Chen, Chae Hyon Lee, Robert Whitfield, and Darren N. Nesbeth

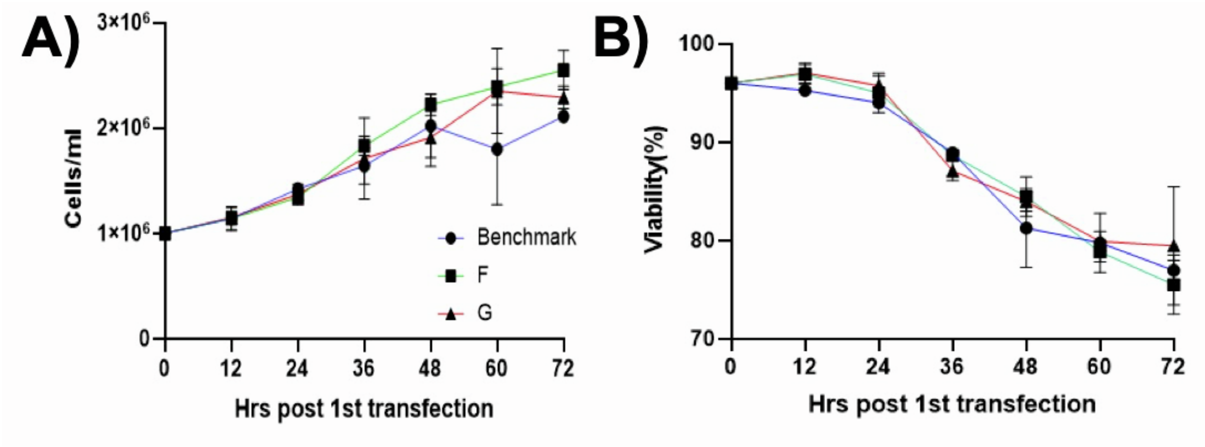

Figure S1. Growth and viability of HEK293F cells after T1 transfection for chronologies Bmk, F and G. From the T1 transfection (Figure 1.3) of chronologies Bmk, F and G, HEK293F cells were analysed with respect to A) Viable cell density (Cells/mL) and B) Viability (%). Error bars represent standard deviation from two biological repeats. Symbol key within plot A also applies to plot B. For transfections chronologies F and G, a second transfection, T2, was performed 20 hours post T1.

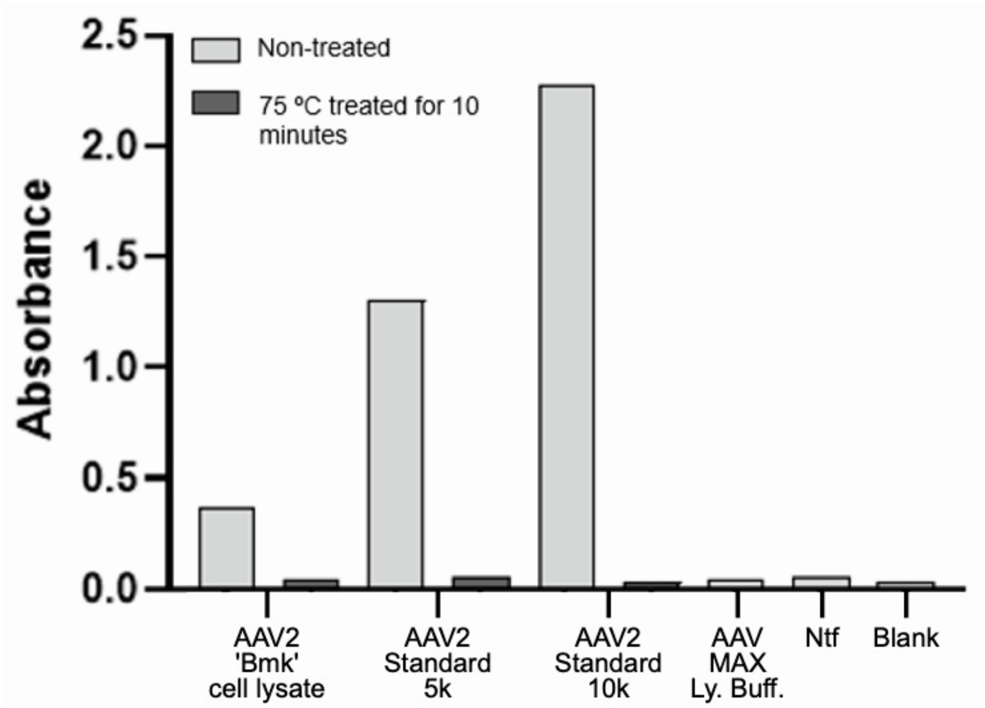

Figure S2. Temperature sensitivity of ELISA signal arising from samples containing AAV2 capids. Samples from benchmark ('Bmk') transfection of HEK293 cells and of standard AAV2 material (Progen, 66V021) were analysed using a Genscript 'AAV2 Titer Capsid ELISA Kit'. Using Sample dilution buffer (Genscript, G1-60) from the ELISA kit as diluent, the cell lysate sample was diluted 1:625 and the AAV standard sample diluted 1:5,000 ('5k') or 1:10,000 ('10k'). Gibco™ AAV-MAX Lysis Buffer (Fisher Scientific, 17331899), lysate from untransfected cells ('Ntf'), and the Sample dilution buffer ('Blank') (Genscript, G1-60), were analysed as controls. All samples were either non-treated (light gray) or placed in a hot block (Bio-Rad, model T100) set at 75°C for 10 minutes (dark gray). Procedures performed in singlet.
